# Chemokine CCL2 and its receptor CCR2 are increased in the hippocampus following pilocarpine-induced status epilepticus

**DOI:** 10.1186/1742-2094-6-40

**Published:** 2009-12-24

**Authors:** Maira L Foresti, Gabriel M Arisi, Khurshed Katki, Andres Montañez, Russell M Sanchez, Lee A Shapiro

**Affiliations:** 1 Department of Neurosurgery, Scott and White Hospital, Temple, TX 76503, USA; 2 Central Texas Veterans Health System, Temple, TX 76504, USA; 3 Department of Surgery, Texas A&M University Health Sciences Center, College of Medicine, Temple, TX 76504, USA

## Abstract

**Background:**

Neuroinflammation occurs after seizures and is implicated in epileptogenesis. CCR2 is a chemokine receptor for CCL2 and their interaction mediates monocyte infiltration in the neuroinflammatory cascade triggered in different brain pathologies. In this work CCR2 and CCL2 expression were examined following status epilepticus (SE) induced by pilocarpine injection.

**Methods:**

SE was induced by pilocarpine injection. Control rats were injected with saline instead of pilocarpine. Five days after SE, CCR2 staining in neurons and glial cells was examined using imunohistochemical analyses. The number of CCR2 positive cells was determined using stereology probes in the hippocampus. CCL2 expression in the hippocampus was examined by molecular assay.

**Results:**

Increased CCR2 was observed in the hippocampus after SE. Seizures also resulted in alterations to the cell types expressing CCR2. Increased numbers of neurons that expressed CCR2 was observed following SE. Microglial cells were more closely apposed to the CCR2-labeled cells in SE rats. In addition, rats that experienced SE exhibited CCR2-labeling in populations of hypertrophied astrocytes, especially in CA1 and dentate gyrus. These CCR2+ astroctytes were not observed in control rats. Examination of CCL2 expression showed that it was elevated in the hippocampus following SE.

**Conclusion:**

The data show that CCR2 and CCL2 are up-regulated in the hippocampus after pilocarpine-induced SE. Seizures also result in changes to CCR2 receptor expression in neurons and astrocytes. These changes might be involved in detrimental neuroplasticity and neuroinflammatory changes that occur following seizures.

## Background

Chemokines are chemotactic cytokines that direct the migration of cells that express the appropriate chemokine receptor. Chemokine C-C motif ligand 2 (CCL2) is a potent attractant protein for monocytes, therefore being previously denominated monocyte chemoattractant protein-1 (MCP-1). The biological effects of CCL2 are mediated via interactions with its receptor, chemokine C-C motif receptor 2 (CCR2). CCR2 is a G protein-coupled receptor and has also been designated as CD192, CC-CKR-2; CKR2; CMKBR2; MCP-1-R. Upon binding to CCR2, CCL2 regulates the migration and infiltration of monocytes, T-lymphocytes and natural killer cells to regions of inflammation [1-3]. Studies using CCL2 and CCR2 knockout mice have shown that this ligand-receptor complex is involved in mononuclear cell infiltration at sites of inflammation [4-7].

In the CNS of several different species including humans, CCR2 expression has been demonstrated in endothelial cells, astrocytes, microglia and neurons [8-14]. In addition, CCL2 and CCR2 expressing cells are observed in multiple brain regions including the hippocampus [11,12] and the expression patterns of CCR2 are altered in human neuropathological conditions. These conditions include: multiple sclerosis [15,16], HIV encephalopathy [17,18], Alzheimer's disease [19,20] and epilepsy [21]. Thus, CCL2 and CCR2 are expressed by several cell types throughout the CNS and this expression is altered in conditions of neuroinflammation.

Neuroinflammation occurs after seizures and may contribute to a pro-epileptogenic neural system [22,23]. This is especially true in the hippocampus, where inflammation has been shown to contribute to neuroplastic changes that may have detrimental consequences [24,25]. However, alterations to the expression patterns of CCL2 and CCR2 have not been examined following status epilepticus (SE) in a rat experimental model. Such data could contribute greatly to our understanding of this component of the neuroinflammatory response following an initial precipitating injury such as SE. Thus, this study investigated whether CCL2 or CCR2 expression is altered in the hippocampus following pilocarpine-induced SE. The results show that both CCR2 and CCL2 are increased in the hippocampus following seizures. In addition, it was observed that seizures result in neuroplastic changes to the cell numbers and types that express CCR2.

## Methods

All experimental procedures were approved by the IACUC of the Texas A&M University Health Science Center and the Scott & White Hospital. Animals were housed 2 per cage and maintained in a 12 hour light-dark cycle (06:00-18:00) with food and water provided ad libitum.

### Pilocarpine-induced seizures

Adult Sprague Dawley rats (230-235 g) were treated with methyl-scopolamine (1 mg/kg i.p.; Sigma) 30 min before pilocarpine hydrochloride i.p. injection (320 mg/kg; Sigma). Animal behavior was observed and the onset of SE was considered when animals exhibited a Racine class 3 seizure [26], that lasted more than 5 minutes and progressed to uncontrollable stage 5 seizures. Ninety minutes after SE onset diazepam i.p. (10 mg/kg; Sigma) was administrated to stop seizures. Only animals that presented at least stage 5 seizures based on Racine's scale [26] were included in the SE group (n = 10). Control rats (n = 10) received the same treatment but were injected with saline instead of pilocarpine. Five days after seizure induction rats were sacrificed for analysis.

### Immunohistochemistry

For immunohistochemical analysis, animals (n = 4 per group) were deeply anesthetized with Euthasol (pentobarbital sodium and phenytoin sodium; 175 mg/kg, i.p.) and transcardially perfused with 4% paraformaldehyde in phosphate buffer. The brains were removed, postfixed for 24 hours and cryoprotected in sucrose solution. Forty μm thick sections were cut using a cryostat (Microm HM 505 E) and were processed for immunohistochemistry with anti- CCR2 (rabbit anti-CCR2, 1:200; Abcam). The antibody specificity was verified by omitting the primary CCR2 antibody and also by blocking of CCR2 antibody with the CCR2 peptide (Abcam) that was used as an immunogen for the antibody production.

### Double-labeling immunohistochemistry

In order to decipher the cell types that express CCR2, double-labeling immunohistochemistry was performed using the following antibodies combined with CCR2 staining: neuronal nuclei protein (mouse anti-NeuN, 1:1000; Chemicon) that stains neurons; microtubule-associated protein 2 (mouse anti-MAP-2, 1:1000; Millipore), a neuron-specific microtubule protein; glial fibrillary acidic protein (mouse anti-GFAP-Cy3, 1:500; Sigma), a marker of astrocytes; ionized calcium binding adaptor molecule 1 (goat anti-Iba1, 1:500; Abcam) that stains macrophage/microglia and a marker of endothelial cells (mouse anti-ICAM-1, 1:200, R&D Systems). Tissue processing for immunohistochemistry was performed as previously described [27]. Briefly, sections were incubated in 0.5% H2O2 for 30 min and 1% H2O2 for 1 hr, followed by PBS (0.1 M, pH 7.4) rinsing. Nonspecific staining was blocked by incubating the tissue for 1 hr in 3% normal goat serum (Vector Labs), in PBS. Sections were next incubated in primary antibodies, in PBS with 3% normal goat serum and 0.05% Tween-20, rotating for 48 hr, at 4°C. Sections were then washed in PBS and incubated with secondary fluorescent antibodies (Alexa 488 and 555, 1:200; Millipore), rotating at RT for 90 min. The tissue was next rinsed in PBS baths. It should be noted that the anti-GFAP (Sigma) is conjugated to CY3, thus no secondary antibody was used. It should also be noted that for the anti-Iba1 reaction, normal horse serum (3%; Vector Labs) was used instead of normal goat serum. Brain sections were then mounted onto glass slides and coverslips were applied with hard set mounting medium with DAPI (4',6-diamidino-2-phenylindole; Vector Labs), to allow nuclear visualization.

### Confocal images

All immuno-labeling was verified using a laser scanning confocal microscope (Olympus Fluoview 1000). The sequential scanning feature was used to ensure that cross-excitation did not occur. The 3-D confocal images were processed and analyzed with FV10-ASW (version 1.7a) software. To confirm double-labeling of CCR2 with NeuN and GFAP, quantitative co-localization analyses were employed using the Pearson's correlation coefficient, overlap coefficient and co-localization coefficients M1 and M2. Pearson's correlation coefficient is used to describe the correlation of the intensity variation between channels. Overlap coefficient indicates the intersecting volume of the signals. The co-localization coefficients M1 and M2 indicate the contribution of one color channel in the co-localized area. In this study M1 represent a contribution of CCR2, while M2 show a contribution of NeuN or GFAP. In order to generate the co-localization coefficients the regions of interest of each image were selected and the average of the result of each slice that composed the final image was used.

### Stereology of NeuN/CCR2 double-labeled cells

Once staining was verified using the confocal microscope, the number of cells expressing CCR2 was estimated using the optical dissector method [28]. Analysis was performed in different hippocampal regions using a microscope (Nikon Eclipse MU) with a motorized stage connected to a computer running the Stereo Investigator software (MBF Bioscience). Briefly, the dentate gyrus, and hippocampal areas CA1 and CA3 were examined in 5 sections per rat. For the dentate gyrus tracing, molecular and granular layers were delineated together and are referred as ML/GCL in the text. Preliminary population estimates were performed. Based on these population estimates, a counting frame of 100 × 100 μm was distributed in a randomly positioned lattice of 200 × 200 μm. CCR2 positive (CCR2+) cells were counted using as criteria staining in defined round cell bodies. Cellular processes were not considered for stereological counting. In addition to CCR2-labeled cells, the number of CCR2/NeuN double-labeled cells was also counted within the same counting frame. Results were statistically analyzed using a t-test and are presented as density of cells/mm3 ± SEM.

### Distance between Iba1 and CCR2+ cells

The 3-D images of microglia cells stained with Iba1 and CCR2 + cells were captured as described in the section "Confocal images". Using the Olympus FV10-ASW (version 1.7a) software, the CCR2+ cell body and the cell body of the closest Iba1+ cell were traced using the Free Area tool. Using the Line tool, a line was traced between the closest drawn borders of the two cell bodies. The Measurement command calculated the length of the line traced indicating the distance between the CCR2+ cell and microglia in μm. Results were statistically analyzed using Mann-Whitney Rank Sum Test and are presented as mean distance (μm) ± SEM.

### CCL2 assay

Animals (n = 6 per group) were deeply anesthetized and then decapitated. The brain was quickly removed from the skull and the hippocampus and cerebellum were dissected and immediately frozen in methylbutane in dry ice. CCL2 tissue levels were assayed against a standard curve provided by the Milliplex MAP kit (Millipore) and was performed per manufacturer instructions. Briefly, 25 μl of standard, control or a tissue homogenate sample was added to 25 μl assay buffer. To this solution, 25 μl of beads were added and the reaction was allowed to proceed overnight at 4°C. The beads were washed twice with wash buffer and incubated with 25 μl of detection antibody at RT for 2 hrs, followed by the addition of 25 μl of Streptavidin-Phycoerythrin compound and allowed to incubate 30 min at RT. The beads were then washed twice with wash buffer and incubated with 150 μl of Sheath Fluid for 5 min at RT. The fluorescence intensity was measured on the Luminex machine (Millipore, 100 μl, 50 beads per bead set) and the analyte concentration was determined using the BioRad software (BioPlex Manager version 5.0). The assay was run in triplicate to confirm the results. The tissue concentration of CCL2 was normalized to the total protein concentration and presented as pg/μg of total protein ± SEM.

## Results

### Increased CCR2 and CCL2 in the hippocampus after SE

**Figure 1:**
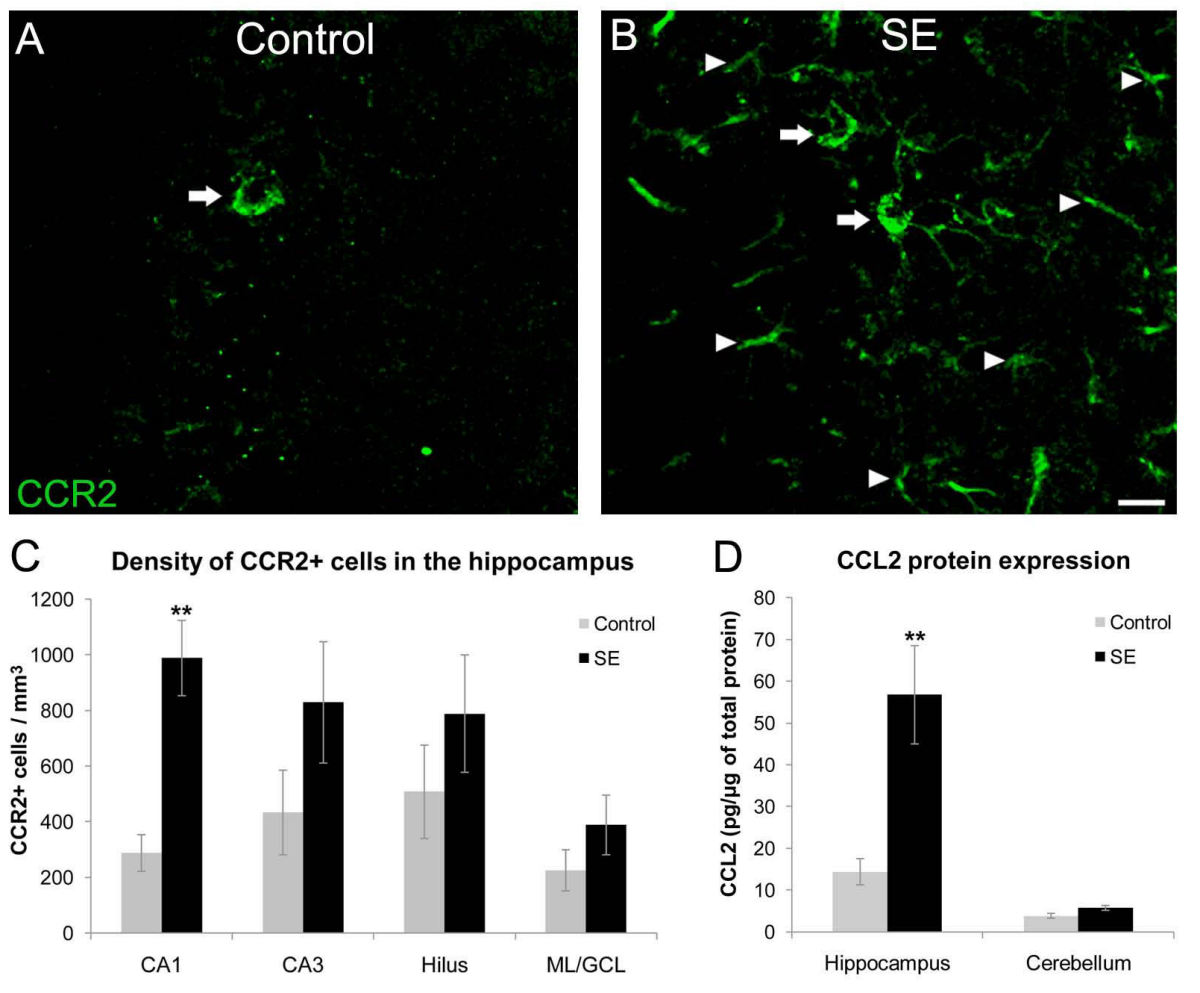
**CCR2 and CCL2 expression in the hippocampus**. Confocal micrographs showing CCR2 staining in control (A) and SE (B) rats. Note that both control and SE rats presented a similar punctate staining pattern of cell bodies (arrows). In addition to cell body staining, SE rats presented numerous isolated processes labeled with CCR2 (arrowheads). (C) Graph of the mean density of CCR2-labeled cell bodies showing that the density of CCR2 labeled cells in SE rats was significantly increased (mean ± SEM, **p < 0.01, t-test) in hippocampal CA1 when compared with control rats. (D) Graph of the mean CCL2 protein expression in the hippocampus showing significantly increased (mean ± SEM, **p < 0.01, t-test) CCL2 expression compared to control rats. Note that there was no change in CCL2 expression in the cerebellum, a region where monocyte infiltration is not expected after SE. Scale bar = 20 μm.

In normal adult rats, the CCR2 immunohistochemical analysis revealed a sparse distribution of cells expressing CCR2 in the hippocampus. In rats that experienced SE, although still sparse, there were significantly more cells labeled with CCR2 (Figure 1). These cells were characterized mainly by punctate staining of cell bodies with limited staining over proximal processes. In addition to increased cell body staining in SE rats, CCR2 labeling was observed in isolated processes that appeared to have either a neuronal or glial phenotype (Figure 1). This pattern of staining was most robust in the dentate gyrus and CA1 region of the hippocampus. Control rats exhibited similar cell body staining, but rarely presented isolated stained processes. Any visible staining was eliminated when the primary antibody was omitted or was conjugated with the CCR2 peptide (data not shown).

Quantitative stereological analysis showed that rats with SE have a significantly increased expression of CCR2 in hippocampal CA1 compared with control rats (p = 0.003, t-test; Figure 1). Results from the cytokine assay also showed a significant increase (p = 0.001) in the expression of CCL2 in the hippocampus of SE rats compared with control animals (Figure 1). There was no change in the CCL2 expression in the cerebellum (Figure 1). This region was used as a control area because it is not greatly affected relative to limbic structures by pilocarpine-induced seizures.

### Neuronal CCR2 expression in the hippocampus after seizures

**Figure 2:**
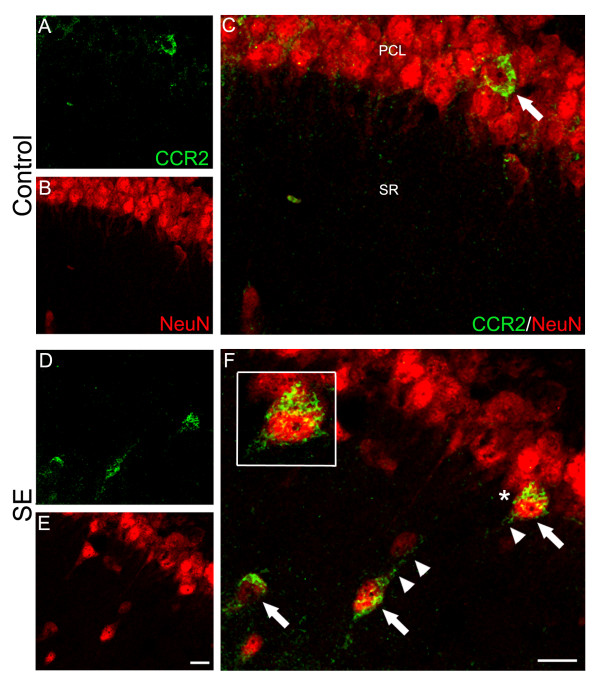
**Confocal micrographs of CCR2 and NeuN double-labeling in hippocampal CA1**. As can be seen in C and F, CCR2 is observed as punctate staining patterns in the peri-neuronal cytoplasm of cell bodies (arrows) in the pyramidal cell layer (PCL) and in stratum radiatum (SR). In addition, the proximal processes of the neurons from SE rats (F) were occasionally observed to label with CCR2 (arrowheads). The cell marked with the asterisk is enlarged in the inset to depict this pattern of labeling (F). Note that compared to control rats (A, C), there was a greater number of CCR2/NeuN double-labeled cells in SE rats (D, F). Scale bar: 20 μm.

**Figure 3:**
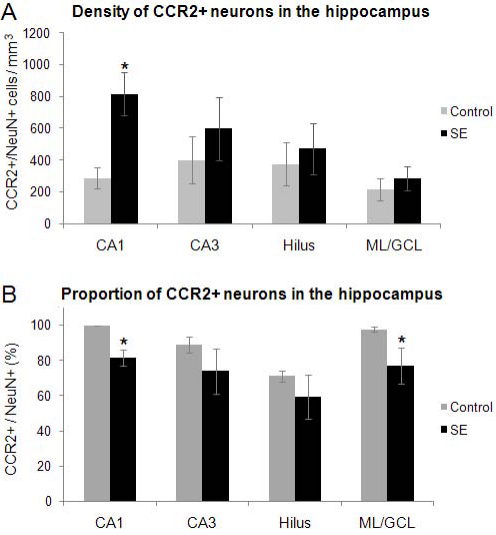
**Stereological analysis of CCR2-labeled neurons in the hippocampus**. (A) The density of CCR2/NeuN double-labeled cells in different regions of the hippocampus. Note that SE rats had a significantly (mean ± SEM, *p < 0.05, t-test) increased density of CCR2/NeuN double-labeled cells in CA1when compared with control rats. Although the graphs appear to show a trend towards an increased density of CCR2/NeuN in the other hippocampal regions, none of them approached significance. (B) Graph depicting the mean proportion of CCR2+ cells that were co-labeled with NeuN. Following SE, the proportion of CCR2-labeled cells that double-label for NeuN was significantly (mean ± SEM, *p < 0.05, t-test) decreased in the molecular layer and granular cell layer (ML/GCL) of the dentate gyrus and CA1 region of the hippocampus. This suggests that other cell types are induced to express CCR2 following seizures.

**Figure 4:**
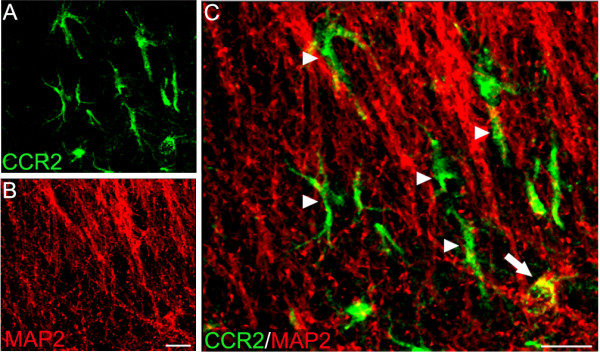
**Confocal micrographs of double-labeling for CCR2 and MAP2 in CA1**. This figure shows CCR2 expression in the cell body that co-localizes with MAP2 (arrow). Alternatively, the isolated CCR2 processes (arrowhead) were not co-labeled with MAP-2, suggesting that they are not neuronal. Scale bar: 20 μm.

Double-labeling immunohistochemistry revealed CCR2+ cells that co-label for NeuN-labeled neurons (Figure 2). The CCR2 staining appeared punctiform and predominantly localized to the surface of the neuron cell body. Since NeuN primarily stains the nucleus of the neuron and to a lesser extent labels the peripheral cytoplasm and proximal processes, the co-localization coefficients were not very robust in these double-labeled preparations (Table 1). Stereological quantification revealed a significant increase in the density of CCR2/NeuN double-labeled cells in CA1 of SE rats (p = 0.029, Figure 3). Further analyses showed that almost all (> 99%) CCR2-expressing cells were double-labeled with NeuN in the molecular and granule cell layers of the dentate gyrus and CA1 region of the hippocampus in control animals. Alternatively, in SE rats, this percentage significantly decreased to approximately 80% of CCR2-labeled cells that co-label with NeuN (p = 0.029; Figure 3). Additional qualitative examination of the neuronal population expressing CCR2 showed a sub-population of MAP-2 labeled cell bodies that also expressed CCR2 (Figure 4). However, MAP-2 labeled neuronal processes were rarely found to double-label with CCR2 (Figure 4).

**Table 1:** Colocalization coefficients.

Region	Double-labeled cells	Animal	**Pearson's Coeff**.	Overlap	Coloc. M1	Coloc. M2
CA1*^a^	CCR2/NeuN	Control	0,28	0,56	0,04	0,04
	CCR2/NeuN	SE	0,29	0,46	0,13	0,13

CA1*^b^	CCR2/GFAP	Control	0,04	0,72	0,00	0,00
	CCR2/GFAP	SE	0,74	0,78	0,74	0,71

DG *^b^	CCR2/GFAP	Control	0,03	0,05	0,00	0,00
	CCR2/GFAP	SE	0,59	0,72	0,61	0,47

**Figure 5:**
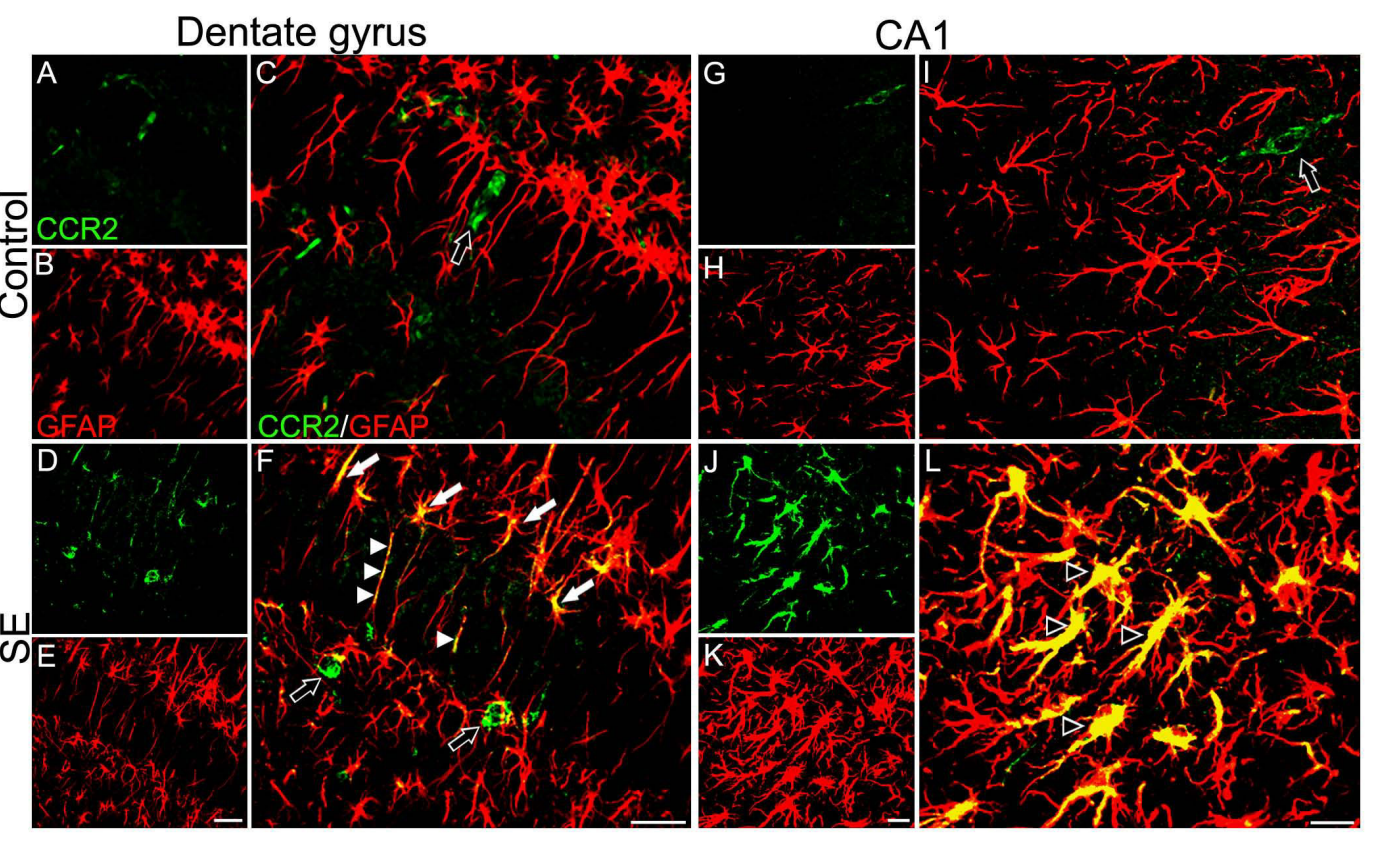
**Confocal micrographs of double-labeling for CCR2 and GFAP in dentate gyrus and CA1**. In the dentate gyrus of SE rats (D-F) the double-labeling is present in the radial processes that extend through the granular cell layer (white arrowheads in F). In addition, a relatively large population of CCR2-labeled astrocytes is oriented toward the hilus (white arrows in F). Alternatively, control rats (A-C) presented CCR2 staining only in the cell body (black arrow in C). This labeling pattern is also observed in SE rats (black arrows in F). In the CA1region of SE rats (J-L) there is a robust co-localization of CCR2 and GFAP in many astrocytes (black arrowheads in L). Note that many of these double-labeled astrocytes have a hypertrophied appearance. In the CA1 region of control rats (G-I), the CCR2 labeling is very limited, being restricted to the cell body (black arrow in I). CCR2 staining in cell bodies (black arrows) of control and SE rats was not observed to co-localize with GFAP. Scale bar: dentate gyrus, 40 μm; CA1, 20 μm.

*a, related to Figure 2. *b, related to Figure 5. DG, dentate gyrus.

### Glial cell expression of CCR2 in the hippocampus after seizures

In addition to the neuronal localization of CCR2, the SE rats had a population of non-neuronal processes labeled for CCR2. These processes were primarily observed in hippocampal area CA1, as well as in the dentate gyrus. Such a robust labeling was not observed in the control rats. Double-labeling immunohistochemistry with anti-GFAP demonstrated that the majority of these CCR2-labeled processes from SE rats were astrocytic (Figure 5). These double-labeled astrocytic processes often appeared hypertrophied (Figure 5). In the dentate gyrus, some of the CCR2-labeled astrocytic processes were the radial glial processes that extend through the granule cell layer and this pattern of labeling was more pronounced in the SE rats. In addition, a relatively robust population of CCR2-labeled astrocytic processes were orientated into the hilus (Figure 5). Such processes were not observed in the control group. The high co-localization coefficients presented in Table 1 confirm the extent of CCR2 and GFAP double-labeling in CA1 and dentate gyrus of SE rats.

### Relationship between microglia and CCR2-expressing cells

**Figure 6:**
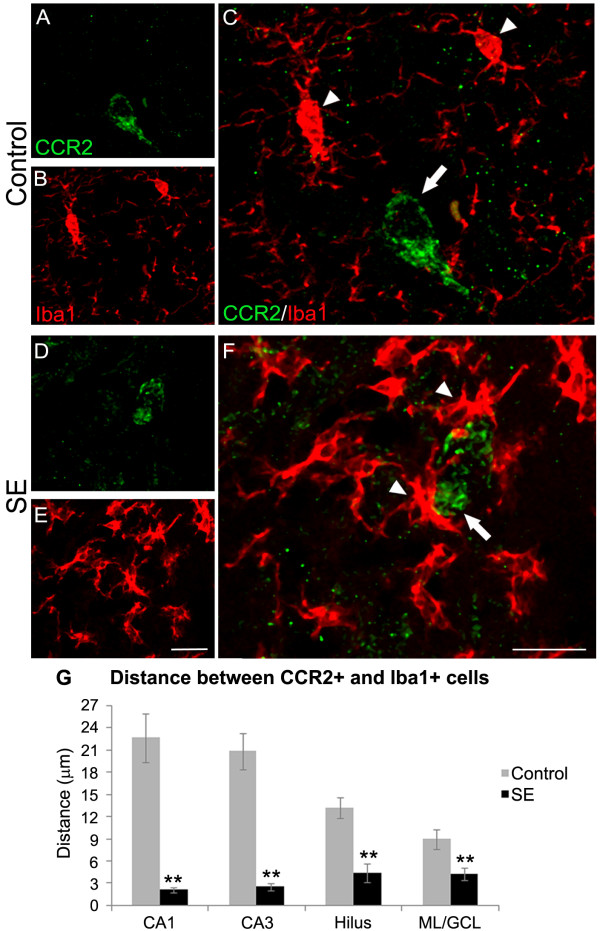
**Confocal micrographs of double-labeling for CCR2 and Iba1 in the hippocampus**. In control rats (A-C), microglia cells (arrowheads) in the hippocampus do not present a clear relationship with CCR2+ cells (arrow). On the other hand, rats that experienced SE (E-F) shows many microglial cells (arrowheads) that appear activated and are closely apposed to CCR2-labeled cells (arrow). (G) Graph of the distance between CCR2+ and Iba1+ cells. Note that compared to controls, SE rats present a significant decrease in the distance between these two cells types, with the mean distance being less than a few micrometers (mean ± SEM, **p ≤ 0.001, Mann-Whitney Rank Sum Test). Scale bar: 20 μm.

Double-labeling with the microglial cell marker Iba1 and CCR2 did not reveal CCR2/Iba-1 double labeling in either the control or SE rats (data not shown). A significant difference was found in the relationship of the proximity between microglial cells and CCR2-labeled cells following SE, such that the microglial cells are more closely apposed to the CCR2-labeled cells. In fact, in some cases, microglial cells appeared to be engulfing the CCR2+ cells (Figure 6). On the other hand, in control animals, there were less microglial cells and these cells did not exhibit an activated morphology that was seen in SE rats. The decrease in the distance between CCR2+ cell and the closest microglia cell was significant in CA1, CA3, hilus, and in the ML/GCL of the dentate gyrus in SE rats when compared with control rats (p ≤ 0.001 in all regions; Figure 6).

### CCR2-expressing cells and endothelial cells

**Figure 7:**
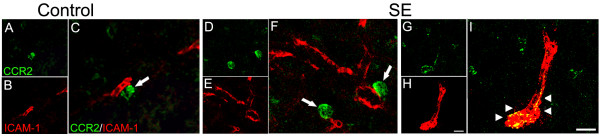
**Confocal micrographs of double-labeling for CCR2 and ICAM-1 in the hippocampus**. In control rats (A-C) some CCR2+ cells (arrow) were identified in the perivascular region (labeled in red). In SE animals (D-F), similar CCR2+ cells in the perivascular region (arrows) seem to be more frequent than in control animals. In addition, co-localization of CCR2 and ICAM-1was occasionally observed in SE animals (arrowheads, G-I). Scale bar: 20 μm.

In addition to the neuronal and glial characterization of CCR2 expressing cells, the double immunohistochemistry for CCR2 and ICAM-1, showed that a small population of CCR2-labeled cells were double-labeled for the endothelial cell marker ICAM-1 in SE rats (Figure 7). Additionally, in the SE rats, CCR2+ cells were more frequently observed juxtaposed with endothelial cells, in the perivascular regions, relative to controls (Figure 7).

## Discussion

The results of the present study show that CCR2 and CCL2 are increased in the hippocampus following pilocarpine-induced seizures. These data also demonstrate that seizures result in selective changes to the cell types that express CCR2. Analysis of the cell types expressing CCR2 shows that SE rats had an increased density of NeuN-labeled neurons expressing CCR2, compared to control rats. However, the fraction of CCR2-labeled cells that double-labeled for NeuN in control rats was almost 100%, compared to about 80% in SE rats. Therefore, in addition to the increased neuronal CCR2 expression, other cell types are induced to express CCR2 following seizures.

It seems that GFAP-labeled astrocytes, more specifically their processes, are a major source of increased CCR2 labeling in the hippocampus following SE. This data is consistent with reports of CCR2 expression by astrocytes in other conditions of neuroinflammation [29]. It is important to note that CCR2 labeling in astrocytic processes was not considered for stereological cell counts and the additional CCR2-labeled cell bodies did not appear to be astrocytes. Therefore, other cell types might contribute to increased cellular expression of CCR2 after seizures. Although no Iba1/CCR2 co-labeling was observed, it is possible that these cells might express CCR2 at different timepoints after injury.

Increased expression of CCL2 in this study is consistent with data from humans, showing elevated levels of CCL2 in tissue from patients with intractable epilepsy [21]. Functionally, increased CCL2 and CCR2 expression in the hippocampus might be a mechanism to activate and recruit inflammatory cells to this injured site after seizures. It is known that that CCL2 binds to CCR2 on brain endothelial cells and contributes to increased brain endothelial permeability [30]. This increased permeability promotes the recruitment of inflammatory cells, such as monocytes and leukocytes at sites of inflammation [31]. Following pilocarpine-induced SE, it was shown that CCR2 is expressed by some endothelial and perivascular cells. These cells partially account for the non-neuronal increase in cellular expression of CCR2 after SE.

The close relationship between activated microglia and CCR2-labeled cells in SE animals suggest that the chemokine-receptor interaction might influence the glial reactivity. Since CCR2-labeled cell bodies presented neuronal morphology it is possible that the microglial activity may be related with neuronal damage following SE.

Through activation of CCR2, the chemokine CCL2 is able to direct the movement of astrocytes [13,32]. The fact that CCR2 expression by astrocytes was robustly observed in CA1 and the dentate gyrus following SE, is consistent with the idea that these regions are typically damaged after prolonged seizures [33-35]. The data showing CCR2 expression by hypertrophied astrocytes in the dentate gyrus following seizures is of particular interest because previous studies have shown astrocyte and microglial activation in the dentate gyrus at the same 5 day timepoint following pilocarpine-induced seizures [27]. In addition, previous studies have shown that after SE, hilar basal dendrites from immature neurons aberrantly grow along an ectopic glial scaffold into the hilus [36]. The astrocytic processes that contribute to the ectopic glial scaffold [36] were observed to be in a similar location as the CCR2-labeled astrocytic processes in this study. It is pertinent to note that the hilar basal dendrites that grow along these astrocytic processes are integrated into an aberrant circuit [37,38] that may facilitate seizure activity [39]. Thus, astrocytic CCR2 may be involved in mediating neuroplastic changes to newly generated neurons and their associated astrocytes following seizures.

Consistent with the notion that CCR2 may play a role in adult neurogenesis are the data showing neural progenitor cells in the adult brain that express CCR2 [40,41]. It is thought that this expression is involved in directing migration of neuronal progenitor cells to sites of neuroinflammation [42,43]. Interestingly, ectopic migration of newborn neurons into the hilus is another seizure-induced neuroplastic change that contributes to a hyper-excitable circuitry [39,44,45]. Thus, it is plausible that increased CCL2 and CCR2 expression in the dentate gyrus are involved in pro-epileptogenic, neurogenic alterations. Future experiments using knockout mice and/or pharmacological manipulations will be needed to test this hypothesis.

## Conclusions

In conclusion, this study shows increased expression of CCL2 and CCR2 in the hippocampus following seizures. The results also demonstrate that CCR2 is preferentially increased in neurons and astrocytic processes after SE. In light of the existing literature suggesting an inflammatory role in epileptogenesis, the data suggest that this chemokine-receptor interaction might be involved in the epileptogenic progression. Future studies should examine the effect of manipulating this chemokine/receptor complex and its effect on seizures.

## Competing interests

The authors declare that they have no competing interests.

## Authors' contributions

MLF helped with pilocarpine injections, animal care and necropsies. She also conducted the immunohistochemistry staining and analyses and drafted the manuscript. GMA carried out all animal experiments and helped with tissue analyses, including statistic analyses of chemokine assay. He also helped with drafting the manuscript. KK conducted the multiplex assays and analysis for CCL2. AM helped with animal experiments and prepared tissues for immunohistochemical procedure. RMS provided intellectual support and ideas for experimental procedures and design. LAS is the principal investigator for this project; he produced the basic project design and directed and coordinated its realization. He also helped in drafting and preparing the manuscript for publication. All authors read and approved the final manuscript.
